# Enhanced fortification of human milk to meet preterm infant nutritional targets

**DOI:** 10.1038/s41390-025-04754-y

**Published:** 2026-01-15

**Authors:** Ting Ting Fu, Carla M. King, Jae H. Kim, Jareen Meinzen-Derr

**Affiliations:** 1 Division of Neonatology, Perinatal Institute, Cincinnati Children’s Hospital Medical Center, Cincinnati, OH USA; 2 Department of Pediatrics, University of Cincinnati College of Medicine, Cincinnati, OH USA; 3 Division of Biostatistics and Epidemiology, Cincinnati Children’s Hospital Medical Center, Cincinnati, OH USA

## Abstract

**Background:**

Many preterm infants need nutrition beyond 24 kcal/oz (standard fortification) to support growth. Individualized targeted fortification improves growth but is labor-intensive. Universal enhanced fortification to presumed 26 kcal/oz for very low birth weight infants was clinically implemented. We determined how often donor breast milk (DBM) and preterm maternal breast milk (MBM) met nutritional targets with enhanced fortification.

**Methods:**

MBM/DBM samples were collected for a prospective cohort study of infants born <1500 g and <33 weeks. Macronutrients were measured using a mid-infrared analyzer. The frequency at which MBM/DBM samples met intake goals (4.8–8.1 g/kg/day fat, 11.6–13.2 g/kg/day carbohydrate, 3.5–4.5 g/kg/day protein) with enhanced and standard fortification was compared.

**Results:**

Among 198 MBM samples and 168 DBM samples, MBM had higher protein, fat, and energy (*p* < 0.0001). Regardless of fortification method, MBM samples met lower and mid-range fat goals more often (*p* < 0.01 and *p* = 0.03, respectively). Collectively, more samples achieved protein targets with enhanced fortification: all samples reached 3.5 and 4 g/kg/day, 56% (147 MBM, 58 DBM) attained 4.5 g/kg/day. With standard fortification, 11% achieved 4 g/kg/day protein; none attained 4.5 g/kg/day.

**Conclusions:**

Enhanced fortification is an efficient method that meets enteral nutrition goals for preterm infants and delivers desired protein intake more consistently.

**Impact:**

- Enhanced fortification is a feasible and efficient alternative to targeted fortification that can also achieve enteral nutrition goals for preterm infants.
- Enhanced fortification delivers desired protein intake more consistently than standard fortification, especially with donor breast milk.
- Regardless of fortification strategy, maternal breast milk is more likely to reach fat intake goals, although additional enrichment may be needed, depending on the fortifier product.

## Introduction

Human milk is the enteral feeding standard for preterm infants, with fortification recommended to meet their nutritional needs.^1,2^ The standard of care is to fortify human milk to 24 kcal/oz (standard fortification) by adding a fixed amount of a multicomponent fortifier agent; however, this strategy relies on assumptions regarding the macronutrient content of human milk, which is often unknown.^2,3^ In practice, many preterm infants need higher caloric recipes to support adequate growth.^2,4–6^ Human milk feedings may either be maternal breast milk (MBM), which is preferred, or pasteurized donor breast milk (DBM), the recommended alternative when MBM is unavailable or insufficient.^7–9^ Unfortunately, DBM is not equivalent to MBM in its nutrient content, which may magnify the need for increased nutritional support.^10,11^

Currently, there is no consensus regarding the optimal method for additional fortification above standard fortification. Due to the variability in human milk composition, there is growing interest in targeted fortification, an individualized process in which human milk nutrient composition is analyzed, and fortifier or modular agents are added to meet nutritional goals. In one of the earliest studies of targeted fortification, every single milk sample required additional enrichment after standard fortification.^12^ Although targeted fortification is associated with greater growth outcomes, due to limitations surrounding reliable human milk analysis, it can be labor-intensive, difficult to implement, and potentially cost-prohibitive.^13–16^

In 2019, our center clinically implemented an enhanced fortification strategy for all very low birth weight (VLBW) infants (born less than 1500 g) to receive human milk fortified to presumed 26 kcal/oz after reaching full enteral volume. This was a proactive strategy that universally provided more nutrition than standard fortification, instead of a reaction to growth faltering. In this study, we ascertained the frequency at which DBM and preterm MBM met recommended nutritional targets with enhanced fortification and in comparison to standard fortification.

## Methods

### Participants

Human milk samples were obtained as part of an ongoing prospective cohort study (Milk Analysis and Growth in Infants of Cincinnati). For this study, infants and mothers were recruited from two level III neonatal intensive care units (NICUs) in Cincinnati, starting in January 2023. The study was approved by the Cincinnati Children’s Hospital Institutional Review Board, University of Cincinnati Office of Clinical Research, and TriHealth Institutional Review Board. Written informed consent for both infant and parental participation was obtained from families.

Infants were eligible if they were born VLBW (less than 1500 g) at gestational age prior to 33 completed weeks; if they attained full feeding volume with enhanced fortification to presumed 26 kcal/oz within the first 30 days of age per usual clinical practice; and if families consented to clinical use of pasteurized donor milk. Infants were excluded if there was a known genetic or congenital anomaly that would limit growth potential or if they were anticipated to transition off donor milk or discharge home within a week of reaching eligibility. Mothers of infants who met these criteria were eligible for parental participation if they were expressing milk.

### Milk samples and analysis

For maternal milk samples, mothers who expressed milk were instructed to create daily pools of all milk that was expressed in each 24-h period. Depending on their milk supply, mothers were instructed to collect samples of their pooled milk up to three times per week.

Per unit routine, both NICUs purchase donor milk for clinical use from the OhioHealth Mothers’ Milk Bank (Columbus, OH). The OhioHealth Mothers’ Milk Bank analyzes and target-pools its milk to provide options of 20 kcal/oz, 22 kcal/oz, and 24 kcal/oz. Upon receiving a shipment of purchased donor milk, a representative bottle from each unique lot number of donor milk was set aside. Trained staff thawed donor milk bottles following unit policy and collected a sample prior to dispensing the remainder of the bottle for clinical use.

To minimize sampling error, mothers and study personnel were taught to agitate milk bottles thoroughly by hand prior to collection, and samples were drawn up using oral feeding syringes. Samples were then stored frozen until batch analysis was performed using a mid-infrared human milk analyzer (Miris, Uppsala, Sweden). Prior to analysis, samples were heated to 40 °C and then homogenized, per the manufacturer’s instructions. For each sample, fat, carbohydrate, protein, and energy content were measured in triplicate and averaged.

### Macronutrient calculations

Reference macronutrient goals selected for the study were based on expert recommendations from the most recent editions of Koletzko et al. and ESPGHAN guidelines on enteral nutrition, as well as evidence from the largest targeted fortification trial to date.^1,13,17^ These goals were 4.8, 6.6, and 8.1 g/kg/day for fat; 11.6, 12.5, and 13.2 g/kg/day for carbohydrate; and 3.5, 4.0, and 4.5 g/kg/day for protein.

Theoretical macronutrient content and the frequency at which fortified samples met macronutrient intake goals were determined assuming a total fluid intake of 150 mL/kg/day and fortification with Similac Human Milk Fortifier Extensively Hydrolyzed Protein (Abbott Nutrition, Abbott Park, IL).^18^ Fortification calculations followed recipes used clinically by the NICUs: For standard fortification to a presumed 24 kcal/oz, 10 mL of fortifier is mixed with 50 mL of human milk, per manufacturer’s direction. For enhanced fortification to a presumed 26 kcal/oz, 15 mL of fortifier is mixed with 50 mL of human milk. Since standard practice in our NICUs is to utilize these ratios regardless of the labeled nutrient content of DBM, calculations for all DBM samples were performed using these ratios as well.

### Statistical analysis

Macronutrient content and proportion of samples meeting target goals were compared using the Mann–Whitney *U* test, Chi-squared test, and Fisher’s Exact test. Cohen’s kappa agreement statistic and McNemar’s test of symmetry were performed to assess concordance between enhanced and standard fortification. To examine the relationship between day of lactation and macronutrient content in MBM samples, separate mixed effects linear regression models were constructed for each macronutrient, with macronutrient concentration as the dependent variable and day of lactation as the fixed effect (independent variable). In these models, maternal participants were included as random effects to account for repeated measures, and a variance components covariance structure was applied. Statistical analysis was completed using SAS version 9.4 (SAS Institute Inc., Cary, NC). Analyses were two-tailed, and statistical significance was set at *p* < 0.05.

## Results

**Fig. 1:**
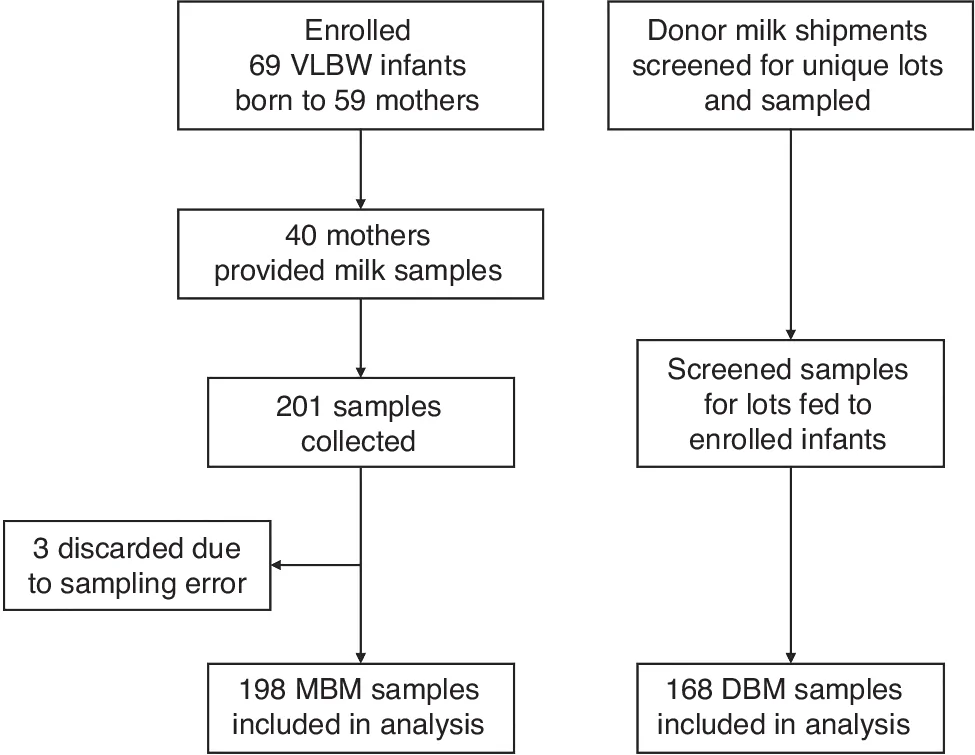
Flowchart of milk sample collection and inclusion.

A total of 366 milk samples were analyzed, with 198 being MBM and 168 being DBM (Fig. 1). Forty different mothers provided milk samples. The median gestational age at time of delivery was 28.2 weeks (interquartile range or IQR 27–30), and the median day of lactation for their samples was 31 (IQR 24–38). Of the 168 DBM samples, 87 were from bottles labeled by the milk bank as 20 kcal/oz, 34 were from 22 kcal/oz bottles, and 47 were from 24 kcal/oz bottles. Table 1 shows a comparison of the measured macronutrient and energy content in DBM and MBM, as well as theoretical content with enhanced and standard fortification. MBM was significantly higher in fat, protein, and total energy (*p* < 0.0001 for all three).

**Table 1:** Macronutrient content of donor milk and preterm maternal milk.

	Donor (*n* = 168)	Maternal (*n* = 198)	*p* value
Fat (g/dL)			
Unfortified	3.6 (3.3–4.1)	4 (3.6–4.5)	<0.0001
Standard fortification	5.6 (5.2–6.2)	6.1 (5.6–6.7)	
Enhanced fortification	5.6 (5.3–6.2)	6.1 (5.6–6.7)	
Carbohydrate (g/dL)			
Unfortified	8 (7.9–8.1)	8 (7.9–8.2)	0.50
Standard fortification	13.8 (13.7–13.9)	13.8 (13.6–14)	
Enhanced fortification	14.4 (14.3–14.6)	14.5 (14.3–14.6)	
Protein (g/dL)			
Unfortified	0.8 (0.7–0.9)	1.1 (0.9–1.2)	<0.0001
Standard fortification	3.5 (3.4–3.7)	3.8 (3.6–4)	
Enhanced fortification	4.4 (4.3–4.5)	4.7 (4.5–4.8)	
Total Energy (kcal/oz)			
Unfortified	20.7 (19.9–22.3)	22.2 (20.9–23.6)	<0.0001
Standard fortification	24.2 (23.5–25.5)	25.4 (24.3–26.5)	
Enhanced fortification	25.5 (24.9–26.7)	26.6 (25.6–27.7)	

Median (interquartile range). *P* values not changed after fortification.

Unfortified content measured by mid-infrared analyzer; fortified content calculated.

Donor milk labeled by milk bank as 20 kcal/oz (*n* = 87) or higher (*n* = 81).

**Fig. 2:**
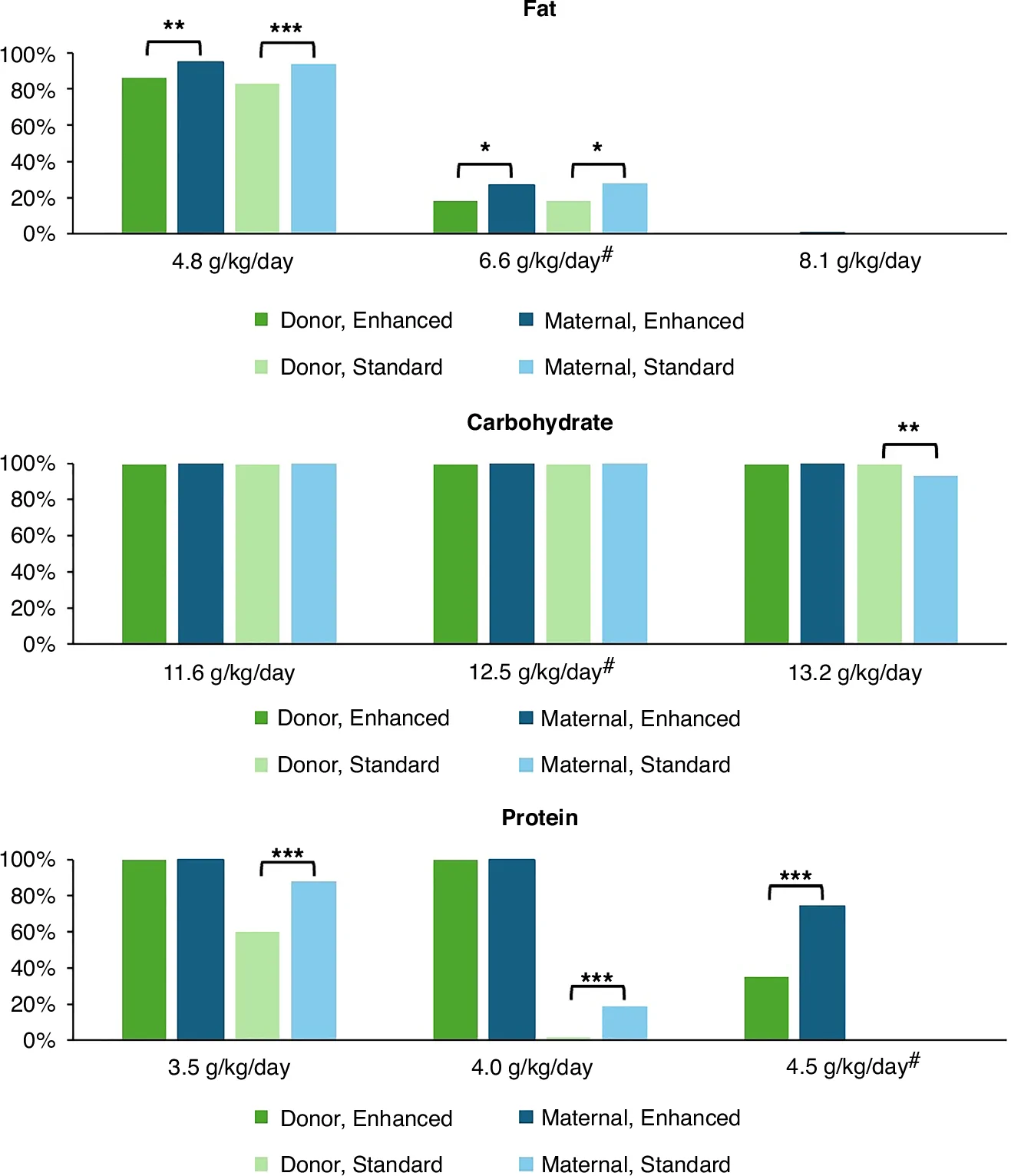
Achievement of macronutrient goals with enhanced and standard fortification. Maternal *n* = 198. Donor *n* = 168. *p < 0.05, **p < 0.01, ***p < 0.001. ^#^Nutrient goals used in the Rochow et al.^13^ targeted fortification randomized clinical trial.

Figure 2 depicts the frequency of DBM and MBM attaining each macronutrient goal after fortification. For fat, MBM samples met the target more often than DBM for both the low-end (94 vs. 83% for standard fortification, *p* < 0.001; 95% vs. 86% for enhanced fortification, *p* < 0.01) and mid-range (28% vs. 18% for standard fortification, *p* = 0.03; 27% vs. 18% for enhanced fortification, *p* = 0.03) goals. Regardless of the fortification method, only 1% of MBM samples and no DBM samples met the highest fat goal. Of note, 25 (83%) of the 30 DBM samples that met the mid-range 6.6 g/kg/day fat goal were from bottles labeled by the originating milk bank as containing either 22 or 24 kcal/oz. Carbohydrate targets were consistently met, especially with enhanced fortification (365 out of 366 samples for all targets).

For protein, all samples met the low-end and mid-range goals with enhanced fortification; 74% MBM vs. 35% DBM samples met the highest protein goal of 4.5 g/kg/day (*p* < 0.0001). Standard fortification did not consistently reach protein intake goals, with 88% MBM vs. 60% DBM reaching 3.5 g/kg/day (*p* < 0.0001), 19% MBM vs. 2% DBM samples attaining 4.0 g/kg/day (*p* < 0.0001), and none from either group achieving 4.5 g/kg/day.

Table 2 shows the agreement between enhanced and standard fortification in achieving the various macronutrient targets. The widest differentials and lowest agreements were observed with the protein targets, but unfortunately, the kappa coefficient could not be determined for any of them, as either all or no samples met the goal for one of the fortification types. This remained true even after stratifying to compare within MBM and DBM subgroups. Samples otherwise demonstrated near-perfect agreement for low-end and mid-range fat and carbohydrate goals (kappa: 0.89–1) and substantial agreement for the highest fat goal (kappa: 0.67). Although the agreement statistic indicated slight concordance for the highest carbohydrate goal (kappa: 0.12) with statistically significant asymmetry, there was 96% agreement between the two strategies.

**Table 2:** Agreement of enhanced vs. standard fortification for meeting macronutrient goals.

		Enhanced	Standard	% Agreement	Kappa (95% CI)	*p* value^a^
Fat	4.8 g/kg/day	334 (91%)	327 (89%)	98.1%	0.89 (0.81, 0.97)	0.008
	6.6 g/kg/day	84 (23%)	85 (23%)	99.7%	0.99 (0.98, 1)	0.34
	8.1 g/kg/day	1 (0%)	2 (1%)	99.7%	0.67 (0.05, 1)	0.34
Carbohydrate	11.6 g/kg/day	365 (100%)	365 (100%)	100%	1 (1, 1)	-
	12.5 g/kg/day	365 (100%)	365 (100%)	100%	1 (1, 1)	-
	13.2 g/kg/day	365 (100%)	351 (96%)	96.2%	0.12 (-0.09, 0.34)	0.0002
Protein	3.5 g/kg/day	366 (100%)	275 (75%)	75.1%	not able to compute	-
	4.0 g/kg/day	366 (100%)	41 (11%)	11.2%	not able to compute	-
	4.5 g/kg/day	205 (56%)	0 (0%)	44%	not able to compute	-

Kappa coefficient and McNemar’s test of symmetry not able to be determined for any protein targets, as either all or no samples met the goal for one of the fortification types. McNemar’s test of symmetry not able to be completed for carbohydrate targets with no discordant pairs.

^a^McNemar test of symmetry. *N* = 366.

The association between day of lactation and macronutrient concentrations is shown in Supplementary Table S1. Protein content decreased by 0.08 g/dL for every 10-day progression in lactation (*p* < 0.0001). While no samples met the highest 4.5 g/kg/day protein goal with standard fortification, 33% (31/94) of MBM samples from the first 30 days of lactation met the 4.0 g/kg/day protein goal with standard fortification, compared to 6% (6/104) of MBM samples expressed after 30 days (*p* < 0.0001).

## Discussion

In this prospective translational study, we report that an enhanced fortification strategy, in which human milk is prepared to a presumed 26 kcal/oz density, can deliver sufficient macronutrients to meet select enteral nutrition goals for preterm infants. Importantly, this strategy delivers desired protein intake more consistently than standard fortification, especially for DBM. When using the Similac brand human milk fortifier agent, though, additional enrichment is needed to reach higher fat intake targets.

The primary benefit of utilizing enhanced fortification and its fixed-dose approach lies in its increased feasibility in implementation, compared to targeted fortification. Targeted fortification for preterm infants is a compelling technique that has demonstrated improved weight gain in randomized trials when significant differences in macronutrient intake are also observed.^13,19^ Targeting protein alone is less consistent in affecting weight velocity but may increase the proportion of fat-free mass accretion.^19–21^ However, accurate measurement, as opposed to assumed reference values, is critical to ensure a meaningful difference in nutrient delivery, and this requires reliable milk analysis performed by trained and capable operators, ideally at a minimum of three times per week for infants receiving MBM.^16,22,23^ For NICUs without access to a human milk analyzer or the budget and staffing to provide the necessary labor, true targeted fortification is unfortunately not possible. Therefore, enhanced fortification provides a practical alternative solution when milk analysis is not achievable.

In comparing enhanced and standard fortification, protein had the greatest difference in macronutrient delivery. Although the kappa agreement coefficient could not be determined due to statistical limitations, there was an appreciable gap between the two methods. Optimizing protein content in human milk feedings has been described in the literature as an intervention that is associated with improved linear growth, fat-free mass accretion, and neurodevelopmental outcomes.^21,24–29^ A wide range of protein content has been reported for DBM, but consistent with our analyses, protein is generally lower in concentration in DBM compared to MBM, closer to 0.9–1.0 g/dL.^11,30–33^ We previously published a protein enrichment strategy specifically for DBM feedings.^4^ It was not surprising that, with standard fortification, only 60% of our DBM samples here met the lowest protein target, and very few did for higher goals, but interestingly, most MBM samples also did not attain the higher goals. While MBM samples from the first 30 days of lactation were more likely to meet the mid-range protein goal, it was still only 33% that did so, and no MBM samples reached the highest protein target. This suggests that enhanced fortification allows for improved consistency of protein delivery across the board, highlighting a key benefit.

While the infant outcomes for this study are not yet available, our units have had significant experience and success utilizing the higher-density recipe and fortification strategy described here. This specific ratio of fortifier to human milk has been utilized in the Greater Cincinnati area NICUs since 2016, and the enhanced fortification strategy has been used clinically since 2019. While we acknowledge this higher-density recipe is an off-label preparation, higher caloric recipes are indeed utilized as needed in the clinical setting to support growth.^4,6^ Any recipe beyond standard fortification using a bovine milk-based product is technically outside published manufacturers’ directions, whether it is achieved through additional fortifier or the addition of formula powder. In addition, liquid fortifier products displace human milk; in our practice, this displacement increases from 17% with standard fortification to 23% with enhanced fortification, but it may be more pronounced with other fortifiers.^34^ Displacement of maternal milk especially is a valid concern but must be balanced with the negative consequences of suboptimal growth. We recognize that enhanced fortification may provide nutrition above recommendations as well. As such, further research is ongoing to investigate prospectively the impact of enhanced fortification on growth and clinical outcomes in VLBW infants, which will offer more insight regarding both risks and benefits and actual macronutrient intake. Additional analyses are also warranted to examine micronutrient intake with these recipes.

Other limitations of this study include the assumptions that factored into our calculations, such as the fluid intake of 150 mL/kg/day. This volume was selected because our regional network of NICUs is protocolized to aim for 160 mL/kg/day as full enteral feeding volume, which in practice means infants receive 150–160 mL/kg/day, with individualized care sometimes leading to fluid restriction to 140 mL/kg/day. We recognize that some institutions may choose higher goal feeding volumes.^35^ We also performed our analysis based on the content of a single manufacturer’s human milk fortifier. The fortifier used by our sites has higher carbohydrate and lower fat content,^18^ which is reflected by the consistent achievement of carbohydrate goals and lower rates of attainment for the higher fat goals. Other multicomponent fortifier products may differ in macronutrient ratios, with higher fat and lower carbohydrate content. Thus, when different fluid intakes are prescribed (either greater or lower) or other fortifiers are used, the results may be different from our reported findings.

Moreover, our DBM samples only reflect the pooling practices of the OhioHealth Mothers’ Milk Bank, specifically, target pooling. This is evidenced by the presence of higher calorie samples as well as the lower end of the interquartile range for energy still measuring close to 20 kcal/oz. Milk banks that do not have access to a human milk analyzer to perform target pooling may rely instead on random pooling, which can yield less consistency in fat delivery, as can varying the number of donors per random pool.^31,32^ Currently, milk analysis and target pooling are not required by the Human Milk Banking Association of North America.^36^

One main strength of our study is the standardized milk collection technique we implemented for milk samples. DBM samples are collected by trained staff prior to any milk removal for clinical use, as opposed to samples of convenience or scavenged remnants from DBM containers or feeding tubes. For MBM samples, maternal participants were given explicit pooling and sampling instructions, including directions to combine each day’s expressed milk into 24-h pools. We acknowledge that 24-h pooling may not be a standardized clinical practice at all institutions, meaning feedings prepared with milk from single pumping sessions throughout a day may yield additional variability; however, using 24-h pooling to minimize diurnal variation allows each MBM sample to represent the mother’s overall stage of lactation. Other strengths of the study include accurate macronutrient analysis with a mid-infrared human milk analyzer and evaluation of a range of recommended macronutrient targets.

In conclusion, enhanced fortification can be an efficient method to increase macronutrient delivery. Compared to standard fortification, enhanced fortification provides more consistent protein provision to meet recommended intake goals for preterm infants. Additional research examining its clinical impact on infants is forthcoming.

## Supplementary information

Supplementary Table S1

## Data Availability

The datasets generated during and/or analyzed during the current study are available from the corresponding author on reasonable request.
