# Supplementary material for: Enhanced fortification of human milk to meet preterm infant nutritional targets
Source: Pediatr Res. 2026 Jan 15;100(3):1359–64. doi: 10.1038/s41390-025-04754-y (PMC13672042; doi:10.1038/s41390-025-04754-y)
Supplement: Supplementary file 1 [file 41390_2025_4754_MOESM1_ESM.pdf]

Supplemental Table 1 – Day of Lactation and Change in Preterm Maternal Milk Macronutrient Content Over Time

Macronutrient	Beta Estimate (95% CI)	p-value
Fat (g/dL)	0.003 (-0.006, 0.012)	0.48
Carbohydrate (g/dL)	-0.0002 (-0.002, 0.002)	0.83
Protein (g/dL)	-0.008 (-0.010, -0.006)	<0.0001
Total Energy (kcal/oz)	-0.005 (-0.031, 0.020)	0.68

Mixed effects linear regression models were conducted with day of lactation as the independent variable, each macronutrient or total energy as the dependent variable, and maternal participant as random effects. Beta estimate represents change in g/dL or kcal/oz per increase in day of lactation. Every 10-day increase in day of lactation was associated with a 0.08 g/dL decrease in protein concentration.
